# Public reasoning about voluntary assisted dying: An analysis of submissions to the Queensland Parliament, Australia

**DOI:** 10.1111/bioe.12777

**Published:** 2020-08-19

**Authors:** David G. Kirchhoffer, Chi‐Wai Lui

**Affiliations:** ^1^ Queensland Bioethics Centre Australian Catholic University Brisbane Australia

**Keywords:** assisted suicide, euthanasia, public, reasoning, reasons, voluntary assisted dying

## Abstract

The use of voluntary assisted dying as an end‐of‐life option has stimulated concerns and debates over the past decades. Although public attitudes towards voluntary assisted dying (including euthanasia and physician‐assisted suicide) are well researched, there has been relatively little study of the different reasons, normative reasoning and rhetorical strategies that people invoke in supporting or contesting voluntary assisted dying in everyday life. Using a mix of computational textual mining techniques, keyword study and qualitative thematic coding to analyse public submissions to a parliamentary inquiry into voluntary assisted dying in Australia, this study critically examines the different reasons, normative reasoning and rhetorical strategies that people invoke in supporting or contesting voluntary assisted dying in everyday life. The analysis identified complex and potentially contradictory ethical principles being invoked on both sides of the debate. These findings deepen our understanding of the moral basis of public reasoning about end‐of‐life matters and will help to inform future discussions on policy and law reform. The findings underscore the importance of sound normative reasoning and the use of caution when interpreting opinion polls to inform policy.

## 1 INTRODUCTION

A growing number of jurisdictions are legalizing voluntary assisted dying (VAD) as an option at the end of life.^1^ However, VAD remains controversial.^2^ VAD refers to the legal administration of a lethal drug to a terminally ill person at the request of that person. Different jurisdictions have adopted different terms, such as ‘physician‐assisted suicide’, ‘medical aid in dying’, ‘voluntary active euthanasia’, or ‘assisted suicide’, to describe this practice. There is also wide variation regarding the arrangement of VAD practice among so‐called ‘assisted dying regimes’.^3^

Findings of polls indicate that the general public across many countries think that VAD should be legal for those with physical conditions that cause unbearable suffering.^4^ Reviews of survey and public‐attitude research over the past two decades in New Zealand^5^ and Australia^6^ confirm that the majority of the public supports the legalization of VAD. In Australia, support for VAD has increased substantially: 85% of respondents in 2017 (up from 11% in 1996) endorsed the idea that doctors give ‘a lethal dose when a patient is hopelessly ill with no chance of recovery and asks for a lethal dose’.^7^

Such quantitative findings, however, should be interpreted with caution. Survey results hinge upon the precise language used to describe the practice and the context of the question asked. Magelssen and colleagues demonstrated and measured such ‘framing effects’ of surveys on attitudes towards VAD by distributing two versions of a questionnaire to Norwegian citizens. They found moderate to large question‐wording and question‐order effects on the replies received.^8^

Furthermore, qualitative research on attitudes towards VAD has revealed a high level of complexity in the VAD debate, based on polarizing social expectations, moral values and concerns in facing death and dying.^9^ A synthesis of the international evidence on attitudes towards VAD identified four recurring themes in the debate: concerns about poor quality of life, desire for a good death, worries about potential abuse of the practice, and the importance of personal witness of unbearable suffering.^10^ Earlier research also highlighted socio‐demographic variables that predict differences in the approval of VAD among individuals and between countries. These variables include the religious context, level of attachment to autonomy, and feeling of vulnerability.^11^ A high level of religiosity and religious affiliation has been found to be negatively associated with permissiveness toward VAD.^12^

Although the kinds of ethical arguments used in the debate about VAD and their determining factors are well known,^13^ there is relatively little literature on the various reasons, normative reasoning and rhetorical strategies that people invoke in supporting or contesting VAD in everyday life. Understanding this reasoning is important if laws are to be developed that go beyond merely responding to ‘polls’, especially in light of research that questions such quantitative methods and reveals the complexity of socioeconomic influences on attitudes.

An opportunity to address this gap arose with the Australian State of Queensland's Parliamentary Inquiry into VAD, to which the public were invited to submit their reasons for supporting or opposing VAD. Using a mixture of computational textual mining techniques, keyword study and qualitative thematic analysis, we analysed a large sample of these public submissions. Our analysis revealed the fundamental themes that characterize the reasoning commonly employed by the general public in debates about VAD, and the kinds of rhetorical strategies and normative argumentation to which these themes lend themselves. We discuss how some of these reasons, rhetorical strategies and normative arguments relate to each other, including possible conflicts, for example reasoning that supports VAD for competent persons versus reasoning that supports ending the life of incompetent persons on the basis of a valid advance health directive. The findings deepen our understanding of the moral basis of public reasoning about VAD and will help to inform future policy reform on end‐of‐life care matters.

## 2 METHOD

### 2.1 Data

Queensland is the second largest and the third most populous state in Australia: it covers over 22% of the total Australian continent and is home to about 20% of Australia's population. The Queensland Government launched a parliamentary inquiry into aged care, end‐of‐life and palliative care, and VAD in November 2018. The inquiry committee tabled the final report to Parliament on 31 March 2020.^14^ As part of the inquiry, the Queensland Parliament invited the general public to provide written submissions to express their opinions on VAD. These submissions by individual Queenslanders (not institutions) are analysed in this study.

### 2.2 Design and data analysis

This study combines computer‐assisted keyword and text mining with more conventional qualitative thematic analysis. The use of keyword and textual mining techniques allows the examination of large volumes of qualitative data, which would be unfeasible with traditional qualitative methods. These techniques have been used to study ethical reasoning about the use of sedation in end‐of‐life care.^15^

For the text mining we used Voyant Tools,^16^ an open‐source, web‐based text analysis application widely used by scholars in the digital humanities.^17^ For the visual representation of the findings of the interpretative analysis we used NodeXL,^18^ which is an open‐source social network analysis software package.

Data analysis consisted of the following five steps.

1. Extraction, conversion and preparation of the submissions for text mining and content analysis. All submissions in support of or in opposition to VAD were grouped together and saved as a ‘Yes’ or ‘No’ document in Microsoft Word, respectively.
2. Three lists of *keywords*, comprising the most frequently used and distinctive words of each of the documents (see Table 1), were identified and prepared using Voyant Tools.
3. *Collocated* words for each keyword were identified using Voyant Tools. Collocated words are defined as those words that occurred frequently (≧10 times) in proximity (5 words before or after) to the keywords in each list.
4. The keyword–collocate word pairs of each list were mapped using NodeXL and laid out in two broad clusters according to their position on VAD. We then examined and manually grouped the keyword–collocate pairs in each list into themes based on prior knowledge of how terms may be linked to particular reasons, reasoning or rhetorical strategies in the literature.
5. Those keywords and collocates that constituted a theme were examined in the context of the submissions using Voyant Tools to validate and refine the researchers' interpretation in step 4.

**TABLE 1:** Lists of keywords identified in the ‘Yes’ and ‘No’ documents

**Frequent common keywords** *
**‘Yes’ document**: *die, suffering, dignity, able, terminal, want, years, day, illness, know*
**‘No’ document**: *euthanasia, palliative, patients, medical, care, doctors, assisted, state, patient, legislation*
**Frequent unique keywords** *
**‘Yes’ document**: *choice, mother, cancer, suffer, quality, wish, having, choose, months, loved, died, allow, away, disease, days, dementia, father, living, individual, option, ones, mum, wishes, longer, allowed, let, friends, access, year, husband, left, unable, bed, body, place, painful, hope, alive*
**‘No’ document**: *society, suicide, vulnerable, human, killing, elderly, good, god, kill, community, government, murder, abuse, children, world, consider, families, provide, better, legal, value, love, needs, Australia, sick, treatment, cases, issues, protect, association, bill, burden, countries, committee, legalised, practitioner, disabled, doctor*
**Distinctive keywords** *
**‘Yes’ document**: *slow, friend, competent, endure, advance, knowing, undignified, indignity, horrible, eat, feed, lung, bowel, saw, exit, eligible, swallow, adult, inhumane, obviously, AHD (Advance Health Directive), chair, decline, qualified, bedridden, begged, deny, endured, rational, torture, excruciating, minutes, mums, operation, tried*
**‘No’ document**: *association, infirm, sanctioned, devalues, AMA (Australian Medical Association), conception, peak, combating, mitigated, crossed, commandments, expendable, foster, intentionally*

* **Frequent common keywords** are those words that appear in the top 100 frequently used words list of both documents, but which have a significantly higher (p ≦ 0.001) relative frequency (per one million words) in one of the documents compared to the other document. **Frequent unique keywords** are those words that appear in the top 100 frequently used words in one document that do not appear in the top 100 of the other document. **Distinctive keywords** are those words with a high TF‐IDF (term frequency‐inverse document frequency) weighting and a relative frequency of use of 100/million or above in each of the documents. The TF‐IDF weighting is a standard measure to evaluate how important a term is to a specific document in a collection or corpus.^19^ As this study has only two documents in its corpus, the keywords identified using this method are words that occur only in one of the documents.

### 2.3 Sampling, data extraction and preparation

The public submission process started on 14 November 2018 and ended on 15 April 2019. The submissions were made either electronically via a web form, or as emailed or handwritten letters. Most submissions were then published on the Inquiry's website, but a submission was not published if it ‘uses unparliamentary language, is potentially incriminating, provides a detailed description of suicide or discloses deeply personal information’.^20^

At the time of writing, the Inquiry had released a total of 4,595 (2,511 paper/email and 2,084 electronic) submissions.^21^ Length ranged from one sentence to several pages (electronic submission: mean=161 words, SD=177.1; paper/email submission: mean=346 words, SD=653.4). Of these, 4,475 (97%) were submissions by individuals, and the remaining 120 submissions were by groups or organisations. Information released included the name and suburb of the submitter and his/her position on VAD (yes or no). This study focuses only on the submissions by individuals.

The first 1,400 submissions (600 paper/email and 800 electronic), or about 24% of all paper/email submissions and 38% of the electronic submissions as released by the Inquiry, were analysed. Submissions were published largely in chronological order. A comparison of the submissions analysed with samples from later submissions found no obvious differences between the two tranches.

A total of 1,119 submissions (395 paper/email and 724 electronic) were included in the analysis; 281 submissions were excluded from the study for discussing issues other than VAD or because of incomplete information, including 81 that failed to indicate whether they were in support of or in opposition to VAD. All submission contents for or against VAD were pasted into a separate ‘Yes’ or ‘No’ Microsoft Word document. The ‘Yes’ and ‘No’ documents were 201,826 words and 53,658 words, respectively. These two documents constituted the ‘corpus’ that was uploaded to Voyant Tools for keyword and collocate analysis, followed by mapping and manual grouping of the keyword–collocate word pairs in NodeXL.

## 3 RESULTS

### 3.1 Frequent common keywords–collocates

**FIGURE 1:**
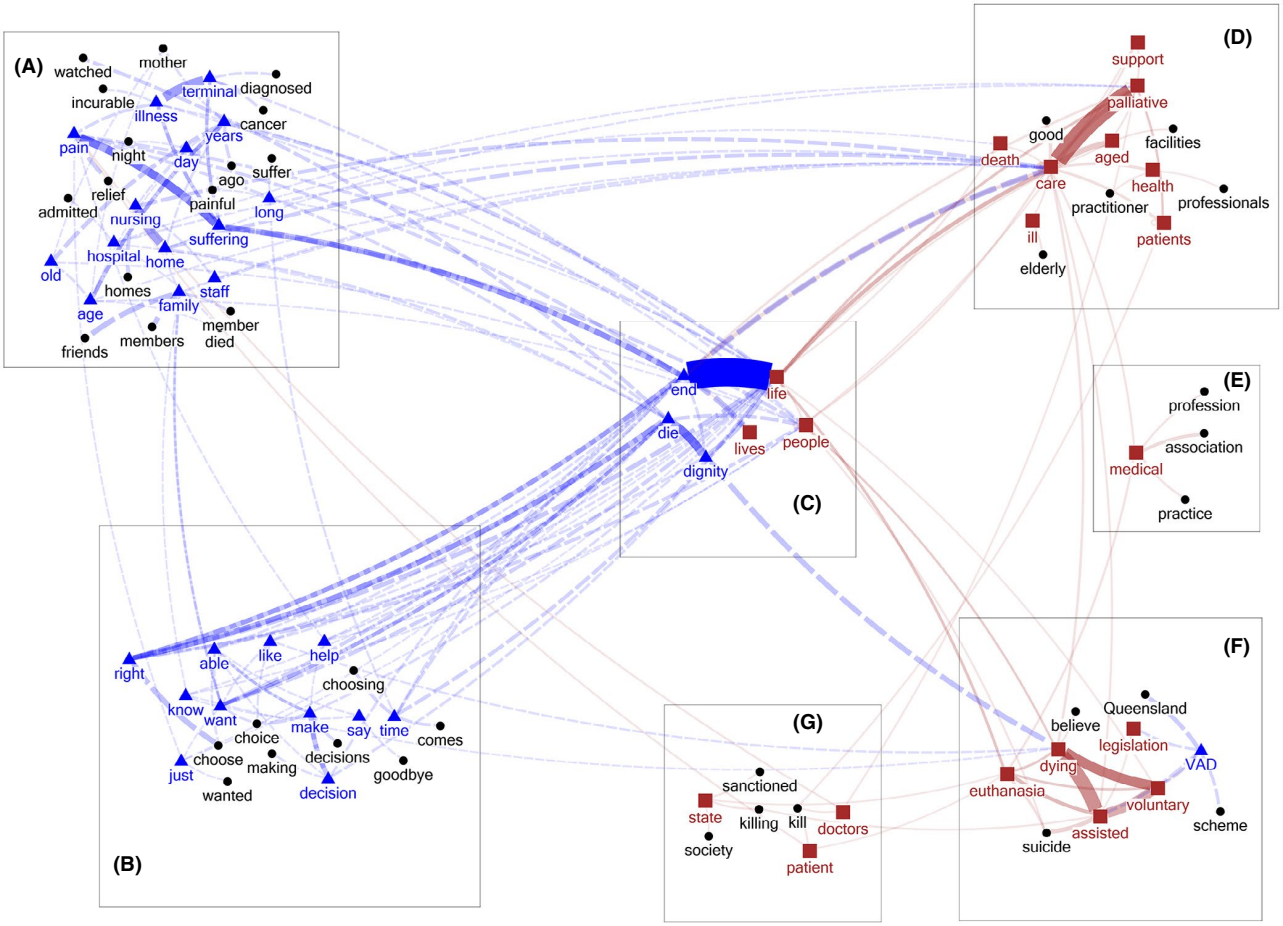
Themes about VAD identified from the list of frequent common keywords–collocates (only word pairs that have co‐occurrences ≧10 times are shown). Words in blue/by a triangle = ‘Yes’ keywords; words in brown/by a square = ‘No’ keywords; words in black/by a circle = collocates. Blue dashed line = ‘Yes’ keyword–collocate pairs; brown solid line = ‘No’ keyword–collocate pairs; the thickness of the lines is proportional to the frequency of the word pairs in the specific document

Frequent common keywords are words found in both documents but that had a significantly higher relative frequency of use in one of them. On examining these keywords and their collocates, we identified a total of seven themes (three supporting and four opposing VAD legalisation), as represented by the clusters A–G in Figure 1.

#### 3.1.1 Theme A: Narrative accounts of witnessing or experiencing pain and suffering

Theme A is characterized by keywords like ‘pain’, ‘suffering’, ‘terminal’, ‘illness’, ‘hospital’, and ‘family’. This theme consists of descriptions of personal experiences and feelings about watching and/or looking after someone (usually family members or friends) who suffered from pain or a terminal illness in various settings. This was verified by examining these keywords and their collocates in context. Examples include:

‘… my father is currently in aged care suffering intractable pain.’

‘It is hard to believe, but that was only the beginning of the most horrific pain and suffering I could ever imagine witnessing.’

‘It was entirely obvious that death was unavoidable, yet nothing was done to move it along so she could be spared the futile pain, suffering and indignity that she went through.’

#### 3.1.2 Theme B: Respect for choice and the right to die

Theme B comprises keywords like ‘right’, ‘want’, ‘able’, ‘make’ and ‘decision’. Broadly, this theme appears to be concerned with personal freedom or autonomy. A closer examination of these words and collocates in context, however, suggests two related reasons that have different implications for reasoning and consequences: (a) the idea that one should respect people's self‐determining choices about when and how they die (i.e., a respect for choice argument); and (b) the idea that people have a ‘right’ to die. As Figure 1 shows, the keyword ‘right’ is not associated with keywords like ‘make’, ‘able’ and ‘want’ that are crucial terms for the respect for choice argument. Rather, ‘right’ is strongly associated with ‘die’ and ‘end’, as in ‘right to die’ and ‘right to end one's life’. Examples include:

‘They are in limbo, suffering, and should be allowed to have a right to choose.’

‘I believe before life gets to this stage we should be able to have the choice to apply to an appointed Body to determine what action should be taken. … they should be able to request an end to their suffering.’

‘I do not want to go into a nursing home and I want to end my life while I still have the mental capacity to say goodbye to my family and friends before ending my life at a certain time.’

‘A person should have a right to make a decision about fundamentally important matters that affect him/her, and a civilised society has a moral obligation to afford those who are suffering the right to choose a dignified end.’

#### 3.1.3 Theme C: Dying with dignity

Theme C is clustered around the three keywords ‘end’, ‘die’ and ‘dignity’. Dignity has long been a key concept in the debate. What is important about our finding is that ‘dignity’ language was used *significantly* more frequently by those supporting VAD than by those opposing it. Analysis in context reveals a concern with the importance of the preservation of dignity at the end of life by allowing access to VAD:

‘If I ever get to the stage where my life becomes too painful to live I would like the option to die with dignity and in a way and a time of my choosing, rather than a slow, painful and costly death which would be worse for both me and my family members.’

‘I do not want to go the way my mother did. A vegetable lying in bed waiting to die, but I want to die with dignity.’

‘If there is no hope of getting better and leading any sort of life then we need legislation to help us die with some dignity….’

It seems that dignity here has to do with maintaining self‐respect and/or not being subjected to humiliating circumstances or treatment rather than with the inherent worth of being human. The latter understanding of dignity would more typically be employed in opposing arguments (see Theme I: Sanctity of life, below).

#### 3.1.4 Theme D: Palliative care is effective

Theme D is identified in the ‘No’ document and is focused on end‐of‐life care. The strong association between ‘palliative’ and ‘care’ is unsurprising. Opponents of VAD argue that high‐quality palliative care is not adequately available and that this should be supported because it removes the need for VAD:

‘Euthanasia lobbyists often wrongly assert that the alternative in terminal cases is an agonising death, but the truth is that almost all pain can be mitigated with good palliative care.’

‘Almost all dying people I have been involved with, had their symptoms well controlled when good quality palliative care was available.’

‘Death is a natural part of life and if the person is receiving good palliative care there really is no need for euthanasia.’

‘Don't play God. Good palliative care is available.’

#### 3.1.5 Theme E: Incompatible with medicine/medical authority

The cluster of keywords and collocates in Theme E is mostly concerned with the nature of medical practice and official positions of professional bodies, which are considered to be incompatible with the practice of VAD. Some examples of these are shown below:

‘Physicians‐assisted [*sic*] suicide, like euthanasia, is unethical and must be condemned by the medical profession.’

‘Any form of euthanasia, which this is, is not in the interest of the medical profession.’

‘Life is precious and it is not the prerogative of the medical profession to take a life.’

‘The Australian Medical Association is against euthanasia under any circumstances. As medical people, surely they should be listened to as should the World Medical Association, which is also in opposition to euthanasia.’

#### 3.1.6 Theme F: Euthanasia and suicide are morally wrong

The word ‘euthanasia’ and the word pair ‘assisted suicide’ are used significantly more frequently in the ‘No’ document. Many opponents seem to claim that language like ‘voluntary assisted dying’ is euphemistic and hides the ‘true nature’ of the practice as intrinsically morally wrong:

‘… it is arguable that a move away from the term euthanasia to voluntary assisted dying may be seen as an attempt to provide somewhat of a veil over the latter term to assist its acceptance with the community.’

‘I personally only see one difference between [a]ssisted suicide (euthanasia) and suicide and that is, [in] assisted suicide a medical practitioner gives you the drug instead of you finding your own way to end your life.’

‘In some countries now children are able to select euthanasia and I have read about others which virtually have a ‘mobile service for euthanasia’.’

#### 3.1.7 Theme G: State‐sanctioned killing

Theme G similarly insists on the moral wrongness of the act of VAD as an unjustifiable act of killing. However, here the concern is more with the consequences of legalizing such an act, effectively state‐sanctioned killing by doctors. Some examples of these are:

‘Like abortion, instead of protecting and assisting the most vulnerable in society, euthanasia would legalise their state‐sanctioned killing.’

‘I believe the right to live is the only right and it is a very dangerous path to take when all of society is burdened with state sanctioned killing.’

‘Please don't approve any bill to introduce state killing of those who think they are unwanted.’

### 3.2 Frequent unique keywords–collocates

**FIGURE 2:**
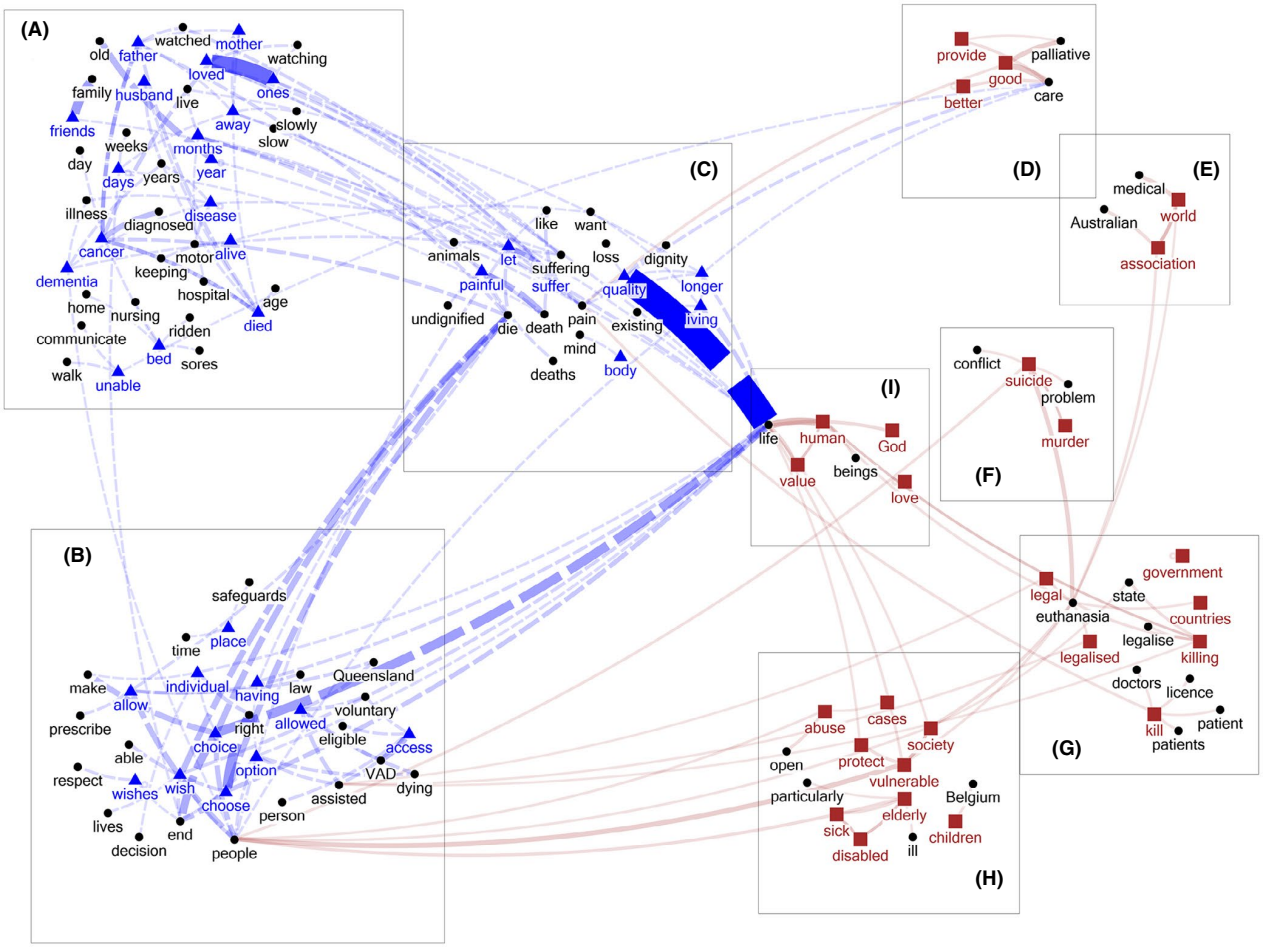
Themes about VAD identified from the list of frequent unique keywords–collocates (only word pairs that have co‐occurrences ≧10 times are shown). Words in blue/by a triangle = 'Yes' keywords; words in brown/by a square = 'No' keywords; words in black/by a circle = collocates. Blue dashed line = 'Yes' keyword–collocate pairs; brown solid line = 'No' keyword–collocate pairs; the thickness of the lines is proportional to the frequency of the word pairs in the specific document

Frequent unique keywords are words found in the top 100 frequently used words of one of the documents but not in the top 100 list of the other document. We identified a total of nine themes (three supporting and six against VAD) (Figure 2). Seven of these were identical to, or extended or refined Themes A to G already examined above. What follows describes this new information and examines two new themes used by opponents (I and H).

Regarding themes supporting VAD, we again found a large cluster of words describing firsthand experiences of suffering (Theme A). ‘Cancer’ and ‘dementia’ are frequently mentioned as causes of the suffering or deterioration. The appearance of ‘dementia’ here is important because it has implications for how ‘voluntariness’ might be understood in legislation (see Theme J: Advance health directives, and the Discussion section below).

Regarding Theme B, which emphasizes respect for choice and/or a ‘right to die’, there emerges the idea of ‘having safeguards in place’. Rhetorically, combining this with ‘respect for choice’ could be an attempt to handle the objection that VAD is against the public interest (Theme G above) and puts vulnerable people at risk (see Theme H below).

Theme C, which emphasizes dying with dignity, includes a new keyword: ‘quality’. This keyword is strongly associated with ‘life’, as in ‘quality of life’. Analysis in context shows that proponents of VAD believe that living longer with suffering, or merely existing with little quality of life, is undignified.

In addition, in Theme C we picked up a new collocated reference to animals. The idea here is that we should be at least as compassionate to human beings as we are to the animals we love: ‘Why can animals be put out of their misery and suffering but humans can't?‘ or ’I only wish my human family could have been afforded the same dignity and kindness that we offer our animals’.

Similar clusters of words corresponding to the four themes against VAD (Themes D, E, F and G) were also identified in the ‘No’ document.

Regarding Theme F (the insistence that VAD is intrinsically morally wrong because it is euthanasia or suicide), we found three important new words. First is the keyword ‘murder’, which like ‘euthanasia’ and ‘suicide’ is used to underscore that VAD is morally unjustifiable killing. Of particular interest, however, are the two collocates ‘conflict’ and ‘problem’. In the case of the former, the argument is that assisting suicide is in ‘conflict’ with the values of medicine (see Theme E). The latter, however, has more in common with the public interest themes (i.e. Themes G and H). The argument is that VAD, by weakening traditional protection of human life and the taboos surrounding suicide, may contribute to worsening the problem of suicide in the community: ‘Euthanasia is also utterly counter‐productive to combating Australia's suicide problem‘, and ’It is the opposite of what is required to stem the rising suicide problem in this country’. For context, Queensland has the second highest rate of suicide in Australia.

#### 3.2.1 Theme H: Protection of the vulnerable

This theme comprises keywords like ‘protect’, ‘vulnerable’, ‘abuse’, ‘children’, ‘elderly’ and ‘disabled’. It is concerned about negative impacts of VAD on vulnerable or marginal groups and society's duty of care towards the most vulnerable. Analysis of the use of these words in context reveals strong concerns that legislation may expose these groups to danger and send the wrong message to the community that the life of vulnerable persons is expendable or not worth living. The case of Belgium is frequently cited as an example of the practice of euthanizing children. Examples of this theme include:

‘Legalisation of euthanasia would expose the vulnerable elderly and terminally ill to pressure—real or imagined—to do the ’right thing' and request death so they are not a ‘burden on their family’.’

‘Capacity to consent is not always clear‐cut and vulnerable lives will be at risk.’

‘To offer this type of option to our weakest and most vulnerable is abusive and cruel.’

‘To move towards a culture… which develops ideologies that treat vulnerable humans as expendable is a criminal act.’

#### 3.2.2 Theme I: The inviolability or sanctity of life

The keywords that stand out in this theme are ‘human’, ‘value’, ‘God’ and ‘love’. The theme invokes the ideas that human life has intrinsic value and that VAD violates this:

‘Every life is precious, and we should value it no matter what.’

‘We should seek to alleviate the pain of the chronically ill, help them to believe in their own intrinsic value to those around them, and we should not assist them in ‘taking themselves out of the way’.’

In some cases, the claim of intrinsic value is associated with assertions that God is the sole giver and taker of life and the only authority to decide when life ends. Life is hence sacred: ‘We are not the authors of our lives only our creator God has this right’ or ‘By giving assisted death to a person in pain or sorrow, we are actually plunging them into even greater pain because they leave this life offending God, taking away God's right over the death of his creature’.

This is the only theme that contains explicit use of religious language or claims.

### 3.3 Distinctive keywords–collocates

**FIGURE 3:**
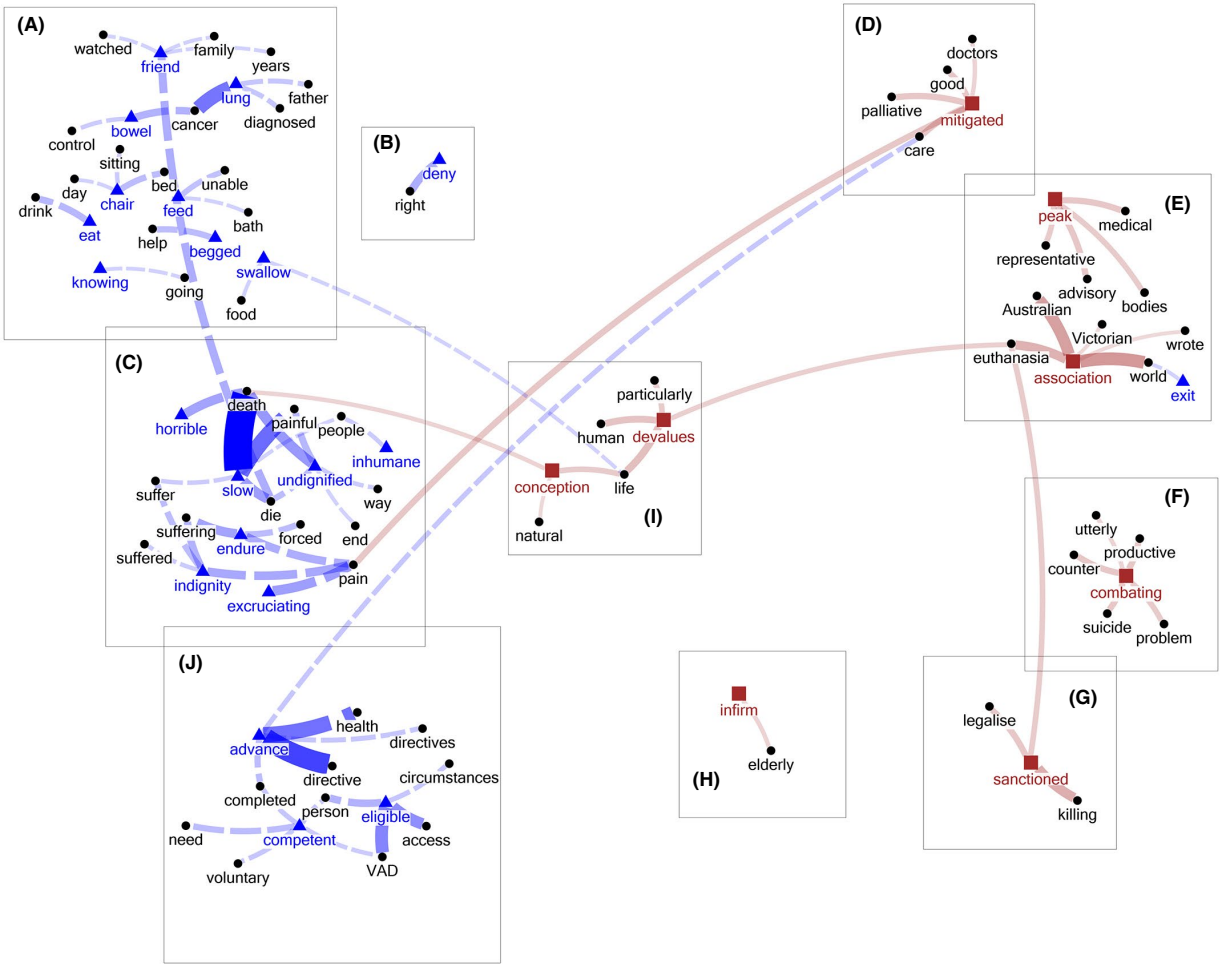
Themes about VAD identified from the list of distinctive keywords–collocates (only word pairs that have co‐occurrences ≧4 times are shown). Words in blue/by a triangle = ‘Yes’ keywords; words in brown/by a square = ‘No’ keywords; words in black/by a circle = collocates. Blue dashed line = ‘Yes’ keyword–collocate pairs; brown solid line = ‘No’ keyword–collocate pairs; the thickness of the lines is proportional to the frequency of the word pairs in the specific document

Distinctive keywords are those words that occur in only one of the documents. Whilst not occurring as frequently as the keywords already discussed, these are included because they are characteristic only of either the ‘Yes’ or the ‘No’ group. One new theme from the ‘Yes’ document was identified (Figure 3).

#### 3.3.1 Theme J: Advance health directives

This theme is associated with the assertion that an advance health directive (AHD) requesting that one's life be ended should be respected, even if one is incompetent at the time that this is done:

‘I believe that every adult Australian should have an Advance Health Directive which states their views should they find themselves in an untenable situation and that these wishes should be carried out by the medical profession.’

‘The request for voluntary assisted dying be made freely and repeatedly by someone with capacity to make such a decision, or alternatively be written into an advance health directive.’

‘I believe if an advance health directive is made while the person has capacity, outlining in what circumstances they want euthanasia, then it should be permitted even after the person has lost capacity.’

## 4 DISCUSSION

### 4.1 A rich variety of reasons

In line with findings of previous research, we found evidence of the four recurring themes in the VAD debate identified by Hendry and colleagues: the importance of personal witness of unbearable suffering, concerns about poor quality of life, desire for a good death, and worries about the potential abuse of the practice.^22^ However, our analysis also identified additional themes used by supporters and opponents of VAD. For those who support VAD, personal witness or direct experience of suffering a terminal illness, a concern about the supposed indignity of suffering and poor quality of life, and respect for individual choice and rights are important themes in their reasoning. For those who oppose it, VAD is an intrinsically immoral practice akin to suicide and murder, violates the intrinsic value of life, undermines the principles and authority of modern medicine, jeopardizes the provision of high‐quality palliative care as the medically and socially appropriate response to suffering, and is against the public interest by permitting state‐sanctioned killing that puts vulnerable groups at risk.

### 4.2 Pathos vs ethos as rhetorical strategies

Different rhetorical strategies seem to be favoured by each side. For the proponents of VAD, subjective and emotionally charged accounts of suffering (Theme A) set the context for and function as a bridge to other arguments, such as demands to die with dignity (Theme C), or to respect choice or a right to die (Theme B). This echoes previous research showing that pathos‐based rhetorical strategies are influential in public debates on VAD.^23^ In contrast, the opponents of VAD tend to employ ethos‐based rhetorical strategies by appealing to the credibility of modern palliative care practice (see Theme D) or professional medical bodies (see Theme E).^24^

It could be argued that pathos is also employed in the opposing view when it insists on the language of suicide, euthanasia and murder rather than VAD, or suggests slippery slopes that will lead to the abuse of the vulnerable and the demise of social mores concerning the inherent value of life. Nonetheless, the pathos strategy seems to be more effective in support of VAD because it offers actual accounts of identifiable people with whom most people can empathize.^25^ Most people do not instinctively identify with vulnerable groups.

### 4.3 Individual vs communal ethical frames

The effectiveness of pathos in the reasoning of supporters may have something to do with an underlying ethical frame of individual self‐interest that has become increasingly prominent in contemporary Western societies.^26^ In this context, pathos rhetoric is effective because people are more likely to personally identify with the claims of respect for choice and a right to die, the maintenance of individual dignity, or avoidance of suffering: the people for whom supporters say VAD is ‘compassionate’ are people like themselves, i.e., free and reasonable (autonomous) individuals. Accordingly, the argument concerning AHDs (Theme J) is not that incompetent people should be euthanized. Rather, it is that the documented wishes of competent (i.e., rational and free) people should be respected if they become incompetent.

In contrast, the opponents of VAD tend towards an ethical commitment to the common good or the public interest. By arguing that VAD is an act of state‐sanctioned killing or suicide, they condemn the practice as a violation of the intrinsic value of life and of traditional obligations of the state to protect vulnerable members of the population. In other words, the debate on VAD legislation can be understood as a clash of individual‐ and community‐level ethical considerations.

It is important to acknowledge that an opponent of VAD could also be arguing from a frame of individual self‐interest, in terms of the fear of society undermining one's desire to live by placing subtle or not so subtle pressures on oneself when one is at the mercy of the ‘system’.

### 4.4 Normative moral reasoning: Deontology and consequentialism

Both sides employ deontological and consequentialist elements in their reasoning, i.e., in how the themes identified are combined to form arguments (Table 2). That said, the individualist vs communal frame seems to be important in determining which principles or consequences are employed in public reasoning, or how these are understood.

**TABLE 2:** Deontological and consequentialist features of public reasoning about VAD

	Deontological	Consequentialist
**Pro‐VAD**	Avoid evil (i.e. suffering) Respect human freedom Protect dignity (do not humiliate) Right to die Euthanize suffering animals	Reduced physical and existential suffering Increased freedom Dignity (as self‐worth) is preserved
**Anti‐VAD**	Avoid evil (i.e. suffering) Respect intrinsic value of life Do not intentionally kill Medicine should cure or comfort Preserve the common good	*Palliative care* will reduce physical and existential suffering and preserve dignity and freedom. *VAD* will lead to: abuse of vulnerable people undermined public interest through state‐sanctioned killing increased suicide rates undermined core values and trust in medicine

#### 4.4.1 Pro‐VAD deontological reasoning

Deontologically, the proponents of VAD employ a mix of the following principles.

1. Unnecessary suffering is evil and should be avoided (Theme A).
2. Human freedom should be respected when there is no substantial harm to others (Theme B, especially with its reference to safeguards in Figure 2).
3. Dignity (understood as self‐worth or freedom from humiliation) should be protected and not violated by ‘forcing’ someone to ‘endure’ ‘horrible’ suffering (see Theme C, especially Figure 3).

Principle (1) is derived from the self‐evident truth that good should be done and evil avoided. Because suffering is bad in itself, it should be avoided. Unnecessary suffering is a moral evil, because it could have been avoided.

Principles (2) and (3) could be used independently of one another. If this happens, there are consequences for how assisted dying is understood. If respect for human freedom is a non‐negotiable principle, then assisted dying can only ever be ‘voluntary’. Even in the case of the use of Advance Health Directives, free choice remains a key presupposition. In contrast, dignity, understood as self‐respect or freedom from humiliation, could be used alone to justify non‐voluntary assistance in dying on the basis of preserving a person from the ‘indignity’ (Theme C, Figure 3) of suffering or loss of quality of life (Theme C, Figure 2). It is not clear from our study whether this distinction and its implications are well understood in public reasoning.

The following two additional principles employed in the public submissions may pose challenges to the three principles already examined above.

- Human beings have a ‘right to die’ (Theme B).
- Compassion demands that we should end suffering by euthanasia, as we already do in the case of animals (Theme C in Figure 2).

The distinction between (2) respect for choice and (4) a ‘right’ to die is important, as the two have different philosophical and legal implications. If one recognizes a right, then it follows that a society has an obligation to ensure that the right is met. Most jurisdictions do not recognize a right to die, but rather the idea that when certain criteria are met (doctors' assessments and so on), a patient is permitted to request to have their life ended. VAD is understood as a justifiable exception to the commitment of governments to protect life.^27^ The claim of a right to die thus puts additional pressure on society not merely to permit the exception but also to provide the means to die when and how one chooses, regardless of the circumstances. It is not clear from our study whether this distinction and its consequences are well understood by the public or whether there is generally a conflation of the two principles.

The appearance of (5) is interesting because it is arguably at odds with (2) and (3), as animals do not have a choice in the matter. They are euthanized because there is no instrumental or existential value in their suffering. This is, however, not the case for human beings, for whom free choice and the desire to be treated with respect and not to be humiliated are both integral to existential meaning‐making. Permitting (5) as the sole reason for VAD may undermine the claims of free choice or respect for dignity and open the door to legitimizing non‐voluntary or even involuntary euthanasia. It is not clear that those who invoke this example are aware of such implications.

#### 4.4.2 Pro‐VAD consequentialist reasoning

From a consequentialist standpoint, the pro‐VAD position is relatively simple: allowing VAD will reduce unnecessary suffering. Yet, how suffering is understood is associated with the principles employed. Whilst physical pain is one kind of suffering (in line with the rhetoric of pathos), the principles of respect for choice and protection of dignity as self‐respect and freedom from humiliation imply that the consequence of permitting VAD would be more respect for human freedom and fewer violations of dignity. These consequences are associated with reduced existential suffering and not necessarily with pain. If the premise is accepted that reducing existential suffering arising from perceived lack of control or fear of humiliation is legitimate as the sole reason for VAD, even in the absence of physical pain, then this potentially broadens the range of circumstances in which VAD would be permissible, including, for example, people experiencing psychological distress.

#### 4.4.3 Anti‐VAD deontological reasoning

For those against VAD, deontological principles include the following.

- Unnecessary suffering is evil and should be avoided.
- The sanctity or intrinsic value of human life must be respected (Theme I).
- Intentional killing, like suicide and murder, is morally wrong (Theme F).
- Medicine must cure or comfort, and never intentionally kill (Theme E).
- Government has a duty to preserve the common good (Theme G) and to protect the vulnerable (Theme H).

Like proponents of VAD, opponents believe that there is a duty to prevent unnecessary suffering, be it physical, psychological or existential (a). They are also likely to agree with the principles of respect for freedom and protection from humiliation, i.e., (2) and (3) above. For opponents of VAD, however, the latter cannot be applied in ways that violate (a)–(e).

The principles used by the public in arguing against VAD are more coherent when used together, with fewer conflicts between principles if different combinations are used as part of an argument (unlike the possible conflicts outlined above regarding (1)–(5)). The only likely problem is if (b) is overemphasized as an absolute rule, such that life should be preserved at all costs regardless of whether there is a proportionate benefit or burden in doing so. It is possible that some members of the public hold this view. Similarly, some proponents of VAD may believe that this rule is the cause of the suffering they wish to end. Neither of these is consistent with mainstream religious or professional medical views, or the current law in Queensland, which allows a person to refuse or withdraw from their own treatment.

#### 4.4.4 Anti‐VAD consequentialist reasoning

From a consequentialist standpoint, opponents appeal to the empirical credibility of the effectiveness of high‐quality palliative care (Theme D) to address the problem of suffering adequately whilst not violating (b)–(e). Against VAD, they argue that ignoring (b)–(e) will result in the abuse of vulnerable people (Theme H), in state‐sanctioned killing undermining the public interest or common good (Theme G), in worsening or at least not helping the suicide problem (Theme F), and in undermining the core values of the medical profession, thereby reducing public trust in the healthcare system that is already under pressure to deliver high‐quality healthcare to all Queenslanders (e.g. Figure 2, Theme E reference to the suicide problem). Whilst the deontological claims require no empirical evidence to support them, the consequentialist ones do. Opponents do make some references to examples, for instance euthanasia of children in Belgium, to support their consequentialist claims. The veracity of the evidence is beyond the scope of the present study.

### 4.5 Religion less important

In contrast to previous findings regarding the importance of religion in opposing VAD,^28^ our research suggests that the religion factor is less important than other factors in the debate in Queensland, at least as far as *public reasons* go. ‘God’ is the only religious terminology that made it into the keyword list (with an appearance of 84 times, or a rank of 30 among the top 100 most frequently used words in the ‘No’ document, versus 42 times, or a rank of 360 in the ‘Yes’ document). This may reflect the increasing secularization of Australian society and the trend of medicalization of end‐of‐life matters over the past decade. But it could also be the case that theological values or arguments may have greater resonance if they are packed in secular terms.^29^ We suggest that further research be conducted to clarify this important issue.

### 4.6 Relationship between legalisation, trust and medicine requires further investigation

Finally, our findings have shed some light on the relationship between VAD legalisation, trust and medicine, which has long been a matter of contention. One frequently used argument against VAD is that legalizing the practice will threaten or undermine trust in the medical profession.^30^ There is conflicting evidence regarding this argument: a U.S. study found no evidence that VAD would lower trust in the medical profession,^31^ while a study in Sweden indicated that this would be the case among those who oppose VAD.^32^

As reported above, our findings revealed that opponents of VAD are worried that legalisation of the practice undermines the principles and authority of modern medicine, and that they tend to appeal to the credibility of professional medical bodies in their argument (Theme E). However, our analysis did not detect ‘trust’ as an important concept. The term ‘trust*’ (where ‘*’ is a wildcard for any ending) appeared only 28 times in the corpus, and it was not identified as a keyword or a collocate in any of the submissions we analysed. To pursue further analysis of the matter of trust in the medical profession requires an in‐depth qualitative content analysis of selected submissions. The scope of such an analysis is beyond the focus of the current article.

## 5 CONCLUSION

This study does not exhaust the possible reasons, arguments or rhetorical strategies used by the public in supporting or opposing VAD. It does, however, help to characterize the main approaches of the two sides: Individualist Pathos vs Communal Ethos. Importantly, it underscores the complexity of reasons and reasoning, and that it is not clear whether this complexity is understood by the public. In particular, in the case of those supporting VAD we see potentially contradictory principles invoked, which, when applied, could have quite different implications for what the resulting ‘assisted dying regime’ might look like. This reiterates the need for caution in relying on surveys of public opinion to make laws concerning VAD. Sound normative reasoning remains vital.

## CONFLICT OF INTEREST

The operations of the Queensland Bioethics Centre at Australian Catholic University are supported by donations from the Roman Catholic Archdiocese of Brisbane and Catholic health and aged care agencies operating in Queensland. In preparing this research, the authors interacted with the Secretariat of the Health, Communities, Disability Services and Domestic and Family Violence Prevention Committee of the Queensland Parliament. The results of the research were submitted to the Committee to inform its own reporting. No funding was received directly for this research. All opinions presented in this manuscript belong to the authors alone, and not any of the above institutions.

## Footnotes

^1^ Cholbi, M. (Ed.) (2017). *Euthanasia and assisted suicide: Global views on choosing to end life*. Santa Barbara, CA: Praeger.

^2^ Sprung, C., Somerville, M., Radbruch, L., Collet, N., Duttge, G., Piva, J., . . . Ely, E. (2018). Physician‐assisted suicide and euthanasia: Emerging issues from a global perspective. *Journal of Palliative Care, 33*(4), 197–203.

^3^ Lewis, P. (2017). Assisted dying regimes. Retrieved from https://webarchive.oireachtas.ie/parliament/media/committees/justice/2017/penny-lewis-assisted-dying-regimes-briefing-paper.pdf.

^4^ The Economist. (2015). Attitudes towards assisted dying; The Economist's poll results. *The Economist, 415,* 19.

^5^ Young, J., Egan, R., Walker, S., Graham‐DeMello, A., & Jackson, C. (2019). The euthanasia debate: Synthesising the evidence on New Zealander's attitudes. *Kotuitui, 14*(1), 1–21.

^6^ Roy Morgan Research Ltd. (2017). It's official: Australians support assisted dying or euthanasia. Retrieved from http://www.roymorgan.com/findings/7373-large-majority-of-australians-in-favour-of-euthanasia-201711100349.

^7^ Ibid.

^8^ Magelssen, M., Supphellen, M., Nortvedt, P., & Materstvedt, L. J. (2016). Attitudes towards assisted dying are influenced by question wording and order: A survey experiment. *BMC Med. Ethics, 17*(24).

^9^ Hendry, M., Pasterfield, D., Lewis, R., Carter, B., Hodgson, D., & Wilkinson, C. (2013). Why do we want the right to die? A systematic review of the international literature on the views of patients, carers and the public on assisted dying. *Palliative Medicine, 27*(1), 13–26; Young et al., op. cit. note 5.

^10^ Hendry, M., et al., op. cit. note 9.

^11^ Verbakel, E., & Jaspers, E. (2010). A comparative study on permissiveness toward euthanasia: Religiosity, slippery slope, autonomy, and death with dignity. *Public Opinion Quarterly, 74*(1), 109–139.

^12^ Danyliv, A., & O'Neill, C. (2015). Attitudes towards legalising physician provided euthanasia in Britain: The role of religion over time. *Social Science and Medicine, 128*, 52–56; Marsala, M. S. (2019). Approval of euthanasia: Differences between cohorts and religion. *SAGE Open, 9*(1).

^13^ Hendry, M., et al., op. cit. note 9; see also Young, R. (2007), *Medically assisted death*. Cambridge, U.K.: Cambridge University Press.

^14^ Health, Communities, Disability Services and Domestic and Family Violence Prevention Committee. (2020) *Report 34, 56th Parliament: Vountary assisted dying*, Queensland Parliament. Retrieved from https://www.parliament.qld.gov.au/Documents/TableOffice/TabledPapers/2020/5620T490.pdf.

^15^ See Seale, C., Raus, K., Bruinsma, S., van der Heide, A., Sterckx, S., Mortier, F., . . . Rietjens, J. (2015). The language of sedation in end‐of‐life care: The ethical reasoning of care providers in three countries. *Health, 19*(4), 339–354.

^16^ Sinclair, S., Rockwell, G., & the Voyant Tools Team. (2012). Voyant Tools (web application). Retrieved from https://voyant‐tools.org/

^17^ Miller, A. (2018). Text mining digital humanities projects: Assessing content analysis capabilities of Voyant Tools. *Journal of Web Librarianship, 12*(3), 169–197.

^18^ Smith, M., Ceni, A., Milic‐Frayling, N., Shneiderman, B., Mendes Rodrigues, E., Leskovec, J., & Dunne, C. (2010). NodeXL: A free and open network overview, discovery and exploration add‐in for Excel 2007/2010/2013/2016, from the Social Media Research Foundation. Retrieved from https://www.smrfoundation.org

^19^ Jurafsky, D., & Martin, J. H. (2008). *Speech and language processing: An introduction to natural language processing, computational linguistics, and speech recognition* (2nd ed.). New York, NY: Prentice Hall.

^20^ Queensland Parliament, *Reports No. 33 and 34, 56th Parliament ‐ Inquiry into aged care, end‐of‐life and palliative care and voluntary assisted dying*. Retrieved from https://www.parliament.qld.gov.au/work‐of‐committees/committees/HCDSDFVPC/inquiries/current‐inquiries/AgedCareEOLPC.

^21^ The final report on VAD as released by the Queensland Government in March 2020 suggested that a total of 4,719 written submissions were received. This paper was drafted with data analysis completed in December 2019. At that time, only 4,595 of the submissions were released to the public. All calculations of percentage mentioned below are based on these 4,595 submissions.

^22^ Hendry et al., op. cit. note 9.

^23^ Duckett, S. (2020). Pathos, death talk and palliative care in the assisted dying debate in Victoria, Australia. *Mortality*, *25*(2), 151–166.

^24^ Rapp, C. (2010). Aristotle's rhetoric. In E. Zalta (Ed.), *Stanford encyclopedia of philosophy* (Spring 2010 edition). Stanford, CA: Stanford University. Retrieved from https://plato.stanford.edu/entries/aristotle‐rhetoric/

^25^ Duckett, S., op. cit. note 22.

^26^ May, T. (2002). *Bioethics in a liberal society: The political framework of bioethics decision Making*. Baltimore, MD: Johns Hopkins University Press.

^27^ Sprung et al., op. cit. note 2.

^28^ Danyliv & O'Neill, op. cit. note 12; Marsala, op. cit. note 12.

^29^ Duckett, S. (2017). Arguing in the public square: Christian voices against assisted dying in Victoria. *Journal for the Academic Study of Religion*, *30*(2), 165–187.

^30^ Sulmasy, D., Travaline, J., Mitchell, L., & Ely, E. (2016). Non‐faith‐based arguments against physician‐assisted suicide and euthanasia. *Linacre Quarterly*, *83*(3), 246–257.

^31^ Hall, M., Trachtenberg, F. , & Dugan, E. (2005). The impact on patient trust of legalising physician aid in dying. *Journal of Medical Ethics*, *31*(12), 693–697.

^32^ Lindblad, A., Löfmark, R., & Lynöe, N. (2009). Would physician‐assisted suicide jeopardize trust in the medical services? An empirical study of attitudes among the general public in Sweden. *Scandinavian Journal of Public Health*, *37*(3), 260–264.

